# A maximum entropy approach for the modelling of car-sharing parking dynamics

**DOI:** 10.1038/s41598-023-30134-9

**Published:** 2023-02-21

**Authors:** Simone Daniotti, Bernardo Monechi, Enrico Ubaldi

**Affiliations:** 1 Complexity Science Hub Vienna, Vienna, 1080 Austria; 2 Vienna University of Technology, Informatics, Vienna, 1040 Austria; 3 Sony CSL, 5 Rue Amyot, 75005 Paris, France; 4 MindEarth, 5 Rue du Manège, 2502 Bienne, Switzerland

**Keywords:** Mathematics and computing, Physics

## Abstract

The science of cities is a relatively new and interdisciplinary topic aimed at studying and characterizing the collective processes that shape the growth and dynamics of urban populations. Amongst other open problems, the forecast of mobility trends in urban spaces is a lively research topic that aims at assisting the design and implementation of efficient transportation policies and inclusive urban planning. To this end, many Machine-Learning models have been put forward to predict mobility patterns. However, most of them are not interpretable -as they build on complex hidden representations of the system configurations- or do not allow for model inspection, thus limiting our understanding of the underlying mechanisms driving the citizen’s daily routines. Here, we tackle this problem by building a fully interpretable statistical model that, incorporating only the minimum number of constraints, can predict different phenomena arising in the city. Using data on the movements of car-sharing vehicles in several Italian cities, we infer a model using the Maximum Entropy (MaxEnt) principle. The model allows for an accurate spatio-temporal prediction of car-sharing vehicles’ presence in different city areas and, thanks to its simple yet general formulation, to precisely perform anomaly detection (e.g., detect strikes and bad weather conditions from car-sharing data only). We compare the forecasting capabilities of our model with different state-of-the-art models explicitly made for time-series forecasting: SARIMA models and Deep Learning Models. We find that MaxEnt models are highly predictive, outperforming SARIMAs while having similar performances of deep Neural Networks - but with advantages of being more interpretable, more flexibile—i.e., they can be applied to different tasks- and being computationally efficient. Our results show that statistical inference might play a fundamental role in building robust and general models describing urban systems phenomena.

## Introduction

In recent years, pressing societal and environmental problems, such as population growth, migration, and climate change, have boosted research on the Science of Cities and the related study of mobility. The parallel growth in the availability of extensive and detailed datasets covering the mobility of individuals at different granular levels has contributed to an increase in the interest of researchers in this field^1,2^. Being multi-disciplinary, Science of Cities studies embrace diverse areas of research. For example, scientists have applied statistical methods to city growth^3^, multi-layer networks to urban resilience^4^, and spatial networks to describe and characterize the structure and the evolution of the phenomena that arise from them^5^. On the modelling side, co-evolution models^6^, and agent-based simulations^7^ have been used to model stylized facts, taking policy-making into account. Moreover, other frameworks, such as ranking dynamics^8^, have been used to study urban environments and their universal laws.

The interplay of different mechanisms, such as the daily routine of individuals and environmental constraints, determines the mobility patterns of individuals diffusing in an urban environment. These, in turn, are regulated by even more fundamental and interrelated phenomena, like wealth, trends, socio-economic disparities, and cultural movements^9–11^. Urban environments are complex systems^12^, and describing their growth and relation with surrounding cities has yet to be fully understood by the scientific community^13^. For instance, when focusing on daily human movement, research has shown that a superposition of decision-making processes working at diverse scales^14^ drives people’s commuting at the intra- and inter-urban level. To capture this complexity, many analytical tools and models have been put in place to understand the patterns of mobility^15–18^.

When trying to characterize human mobility, one open problem is the development of accurate forecasting tools to predict human movement in time and space, a crucial ingredient in urban planning. Indeed, such models would unlock the possibility to compute the optimal capacity of the mobility infrastructure, to correctly assess segregation’s social drivers, or to better design policies aimed at reducing the environmental impact, air pollution, while improving the sustainability of public transport services^19^.

To forecast these kind of aggregated multi-variate time series, different techniques and models have been proposed, from Autoregressive Integrated Moving Average (ARIMA)^20^, Multi-variate non-linear forecasting techniques^21^, Markov Models^22^, up to Deep Learning models^23–25^. However, all these models share a limited capacity to interpret the results; it is difficult to establish the impact of each parameter and each observable on the forecast and to distinguish the relevant signal from irrelevant noise. Therefore, to build a powerful, general, and robust statistical model, we need to understand the critical variables that play a central role in the phenomenon we want to model. To this end, we study urban mobility patterns exploiting the Maximum Entropy (MaxEnt) principle.

The latter builds on information-theoretical grounds to state that the model generating an empirical dataset is the one featuring the most general probability distribution that reproduces the statistically significant observables (e.g., the average value or the standard deviation of a variable). In other words, the MaxEnt modelling framework is a Statistical Inference technique that infers the model’s parameters in a data-driven fashion by maximizing the entropy of its probability distribution, setting a few constraints driven by data observations. The MaxEnt model has already proved to be successful in a wide array of interdisciplinary applications^26^, from biology^27^ to melodic styles^28^.

Here, we propose a Statistical Inference (Maximum Entropy) approach to study and predict urban mobility patterns. To solve it, we need to analyze the dataset we want to study, identify the essential dynamic properties and then model it solving the problem of optimizing the resulting entropy.

The data used in this work represent the $30-minute-binned$ count of cars parked in each district of a city. The cars we focus on are those offered by the major car-sharing service in Italy. The data consist of a multi-variate time series of the activity (i.e., the number of parked cars seen in a given area during a given time bin) in different zones inside the city.

To evaluate our model performance in time-series forecasting, we benchmark it with two state-of-the-art models exploiting statistical and non-linear properties^29,30^, that is, (i) Seasonal Arima^31^, and, (ii) Deep Learning Techniques^32^.

We use MaxEnt inference to obtain the parameters (using gradient ascent algorithm) and reproduce lag-correlations of definite positive time series. We derive a highly predictive model, at least as sophisticated as models that consider non-linear correlations and have more parameters. We also use the obtained statistical model to find extreme events (outliers, such as strikes and bad weather days). As a result, we find the dynamics of cars’ presence in urban areas to be vibrant and complex. We infer the couplings parameters between the activity profiles of different areas and use them to project the cars’ locations in time efficiently.

Since urban systems are notoriously complex and the fundamental causes of the observed mobility patterns are various and interrelated, our methodology is novel in the field since it delivers the most general model under the constraint of reproducing the observed correlations. Moreover, the model reveals to be light in terms of the number of parameters to be trained. It also highlights the importance of linear correlations as compared to non-linear ones.

We organize the rest of this paper as follows. In the "Results" Section, we introduce the data used and the only formalism needed to present the results. We introduce Maximum Entropy models and their formalism, and the optimization algorithm used. Following this, we introduce the formulas to compute an approximated Log-Likelihood. Then, we present the result for multi-variate forecasting of the activity in zones and forecasting of outlier events. Subsequently, the "Discussion" Section will follow. In the Methods section, we describe the data and the variables in use. To determine the essential variables we represent, we carry out a historical analysis and observe the dynamics of contraction and dilation of the time-shifted correlations between different zones. With this, we identify relevant observables, and finally, we define and derive the formulae for the ME model describing our data. In Supplementary Information we add similar results found for the cities of Rome, Florence, and Turin.

## Results

In this section, we introduce the data we analyzed, and our modelling choices. We also test the model forecast and the capacity to detect anomalies, comparing them to state-of-the-art time series algorithms. More in-depth descriptions of the technical details are in the "Methods" section.

### Data description

**Figure 1:**
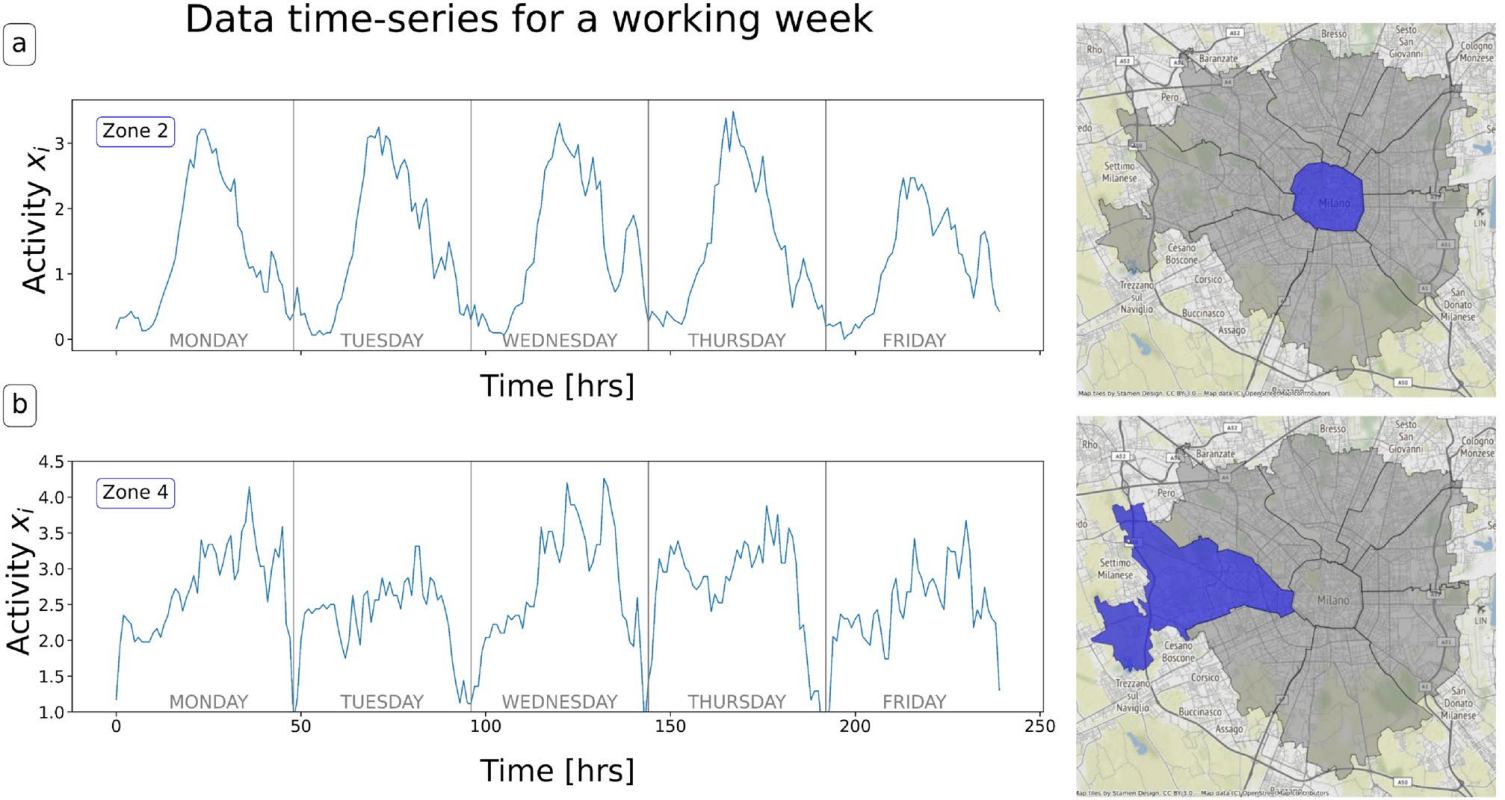
Time series parking activity data for the city center of Milan (**a**) and one of the suburbs (**b**) for a whole week. We can observe that the shapes of the curves and periodicities are different. All the maps have been generated using the python contextily library version 1.2.0.

The dataset contains the position of the vehicles of a major Italian car-sharing service in four Italian cities in 2017 (Turin, Milan, Florence, and Rome). Data were obtained by constantly querying the web APIs of the service provider and record the parking location of each car. This information allows us to establish the origin and destination of each trip. In the "Methods" section, we present the preprocessing procedure we used to wrangle the data and to obtain the time series, which are the input of our models. The preprocessing output is a series of parking events with a location (latitude and longitude) and a starting and ending timestamp. We aggregated these events based on the census tessellation, i.e., the municipality zones inside a city. In the following, we show the case of Milan, which will be our reference city, while we present the remaining cities in the Supplementary Information section. Finally, we count the $x_a(t)$ number of cars parked within area *a* at time *t* for each working day. We normalize $x_a(t)$ (see the "Methods" section for details) and obtain the multivariate time series logging the parking activity of the different municipalities of the city. In Fig. 1, we show an example of the activity in two areas of our dataset covering the Metropolitan City of Milan.

### Maximum entropy principle

The principle of maximum entropy states that the probability distribution best representing the current state of knowledge is the one with the largest entropy, in the context of precisely stated prior data. According to this principle, the distribution with maximal information entropy is the best choice. The principle was first shown by E. T. Jaynes in two papers published in the late fifties; he emphasized a natural correspondence between statistical mechanics and information theory^33,34^.

In particular, Jaynes offered a new and very general rationale to explain why the Gibbsian method of statistical mechanics works. He showed that statistical mechanics, particularly Ensemble Theory, can be seen simply as a particular application of information theory. Hence there is a strict correspondence between the entropy of statistical mechanics and the Shannon information entropy.

Maximum Entropy models have unveiled interesting results over the years for a large variety of systems, like flocking birds^27^, proteins^35^, the brain^36^ and social systems^37^.

We will then implement this approach to define the model of our real-world system in the following sections. A more general introduction to the maximum entropy formalism is out of our scope here, and we refer to the existing literature for details^38–42^.

The probability distribution with Maximum Entropy $P_{ME}$ results from the extreme condition of the so-called *Lagrangian Function*:

1 $$\begin{aligned} {\mathscr {S}} \big [ P \big ] = S \big [ P \big ] + \sum _{k=1}^K \theta _k (\langle O_k \rangle _{P({\underline{X}} )} -\langle O_k \rangle _{obs}), \end{aligned}$$

where

2 $$\begin{aligned} S \big [ P \big ]= - \sum _{{\underline{X}}} P({\underline{X}}) \log (P({\underline{X}})) \end{aligned}$$

is the Shannon Entropy of the probability distribution $P({\underline{X}})$. The maximum of the Lagrangian Function is the maximum of the entropy of the model when it is subject to constraints. Computing the functional-derivative (1) with respect to $P({\underline{X}})$ and equating to zero results in:

3 $$\begin{aligned} P_{me}({\underline{X}}) = \frac{1}{Z({\underline{\theta }})} \exp \Big [- \sum _{k=1}^K \theta _k O_{k}({\underline{X}})\Big ], \end{aligned}$$

where

4 $$\begin{aligned} Z({\underline{\theta }})=\int \limits _\Omega d{\underline{X}} \exp \Big [- \sum _{k=1}^K \theta _k O_{k}({\underline{X}})\Big ] \end{aligned}$$

is the normalization (making a parallel with statistical physics, can be called *Partition Function*). $Z({\underline{\theta }})$ is written as a sum if $\Omega$ is discrete. Hence, the maximum entropy probability distribution is a Boltzmann distribution in the canonical ensemble with Boltzmann constant $K_B=1$, and effective Hamiltonian ${\mathscr {H}}({\underline{X}}) = -\sum _{k=1}^K \theta _k O_{k}({\underline{X}})$.

Note that the minimization of the Lagrangian Function is equivalent to the maximization of the experimental average of the likelihood:

5 $$\begin{aligned} {\mathscr {S}} \big [ P \big ] = \log Z({\underline{\theta }}) - \sum _{k=1}^K \theta _k \langle O_k \rangle _{e} = -\langle \log P_{me} \rangle _{e} = \frac{1}{M} \sum _{m=1}^M \log P({\underline{X}}^{(m)}). \end{aligned}$$

In other words, the $\theta _k$ are chosen by imposing the experimental constraints on entropy or, equivalently, by maximizing the global, experimental likelihood according to a model with the constraints cited above.

Maximum Entropy is the method resulting in the optimal distribution; instead, Maximum Likelihood is the optimization for a parametric model. The optimal parameters of $\theta$ (called *effective couplings*) can be obtained through Maximum Likelihood, but only once one has assumed (by the principle of Maximum Entropy) that the most probable distribution has the form of $P_{ME}$.

Given the generative model probability distribution of configurations $P({\underline{X}} \mid {\underline{\theta }})$ and its corresponding partition function by $\log Z( {\underline{\theta }} )$, the estimator of $\theta$ can be found by maximizing the log-likelihood:

6 $$\begin{aligned} {\mathscr {L}}( {\underline{\theta }} ) = \langle \log (P({\underline{X}} \mid {\underline{\theta }})) \rangle _{data} = - \langle {\mathscr {H}}({\underline{X}}; {\underline{\theta }}) \rangle _{data} - \log Z( {\underline{\theta }} ). \end{aligned}$$

Having chosen the log-likelihood as our cost function, we still need to specify a procedure to maximize it with respect to the parameters.

One common choice widely employed when training energy-based models are Gradient Descent^42^ or its variations: optimization is taken with reference to the gradient direction. Once one has chosen the appropriate cost function ${\mathscr {L}}$, the algorithm calculates the gradient of the cost function concerning the model parameters. The update equation is:

7 $$\begin{aligned} \theta _{ij} \leftarrow \theta _{ij} -\eta _{ij} \frac{\partial {\mathscr {L}}}{\partial \theta _{ij}}. \end{aligned}$$

Typically, the difficulty in this kind of problem is to evaluate $\log Z( {\underline{\theta }} )$ and its derivatives. The reason for this is that the partition function is rarely an exact integral; it can be calculated exactly only in a few cases. However, finding ways to approximate it and compute approximated gradients is still possible.

### Pseudo-log-likelihood (PLL) maximization

Pseudo-likelihood is an alternative method compared to the likelihood function and leads to the exact inference of model parameters within the limit of an infinite number of samples^43,44^. Let us consider the log-likelihood function ${\mathscr {L}}( {\underline{\theta }} ) = \langle \log P({\underline{X}} \mid {\underline{\theta }}) \rangle _{data}$. In some cases, we cannot compute the partition function $Z( {\underline{\theta }} )$. However, it is possible to derive exactly the conditional probability of one component of ${\underline{X}}$ with respect to the others, i.e. $P(X_j | \underline{X_{-j}}, {\underline{\theta }})$ where $\underline{X_{-j}}$ indicates the vector ${\underline{X}}$ without the *j*-th component.

In this case, we can write an approximated likelihood called Pseudo-log-likelihood, which takes the form:

8 $$\begin{aligned} {\mathscr {L}}( {\underline{\theta }} )_{\textit{pseudo}} = \sum _j \langle \log P(X_j | \underline{X_{-j}}, {\underline{\theta }}) \rangle _{data}. \end{aligned}$$

The model we will introduce in this work does not have an explicit form for the partition function, but Eq. (8) and its derivatives can be exactly derived. Thus, the Pseudo-log-likelihood is a convenient cost function for our problem.

### Definition of MaxEnt model

We have seen that the Maximum Entropy inference scheme requires the definition of some observables that are supposed to be relevant to the system under study. Being that the aim is to predict the evolution of the different $x_i(t)$, the most straightforward choice is to consider their correlations.

As a preliminary analysis, we study the correlation between the activity $x_i(t)$ of the most central zone within the city (highlighted in red) and all the other zones with a *shift* in time (i.e. we correlate $x_i(t)$ with $x_j(t-\delta )$ for al $j\ne i$ and some $\delta >0$). The measure of correlation between two vectors *u* and *v* represented is $\frac{u \cdot v}{{||u||}_2 {||v||}_2}$, where ${||.||}_2$ measures the euclidean norm defined in ${\mathbb {R}}^n$ as $\left\| {\varvec{x}} \right\| _2 := \sqrt{x_1^2 + \cdots + x_n^2}$. We see that the areas with significant correlations vary with $\delta$ so that they cluster around the central area for small values and they become peripherals when $\delta \sim 31$. When $\delta \sim 48$, they cluster around the central area again. Peripheral zones show instead the opposite behavior. We perform a historical analysis of time-shifted correlations, observing contraction and dilation with reference to different zones in a one-day periodicity. Due to the correlation dependency on the time shift, we have to include it in the observables used to describe the system. Hence, we chose as observables all the *shifted correlations* between all the couples of zones defined as,

9 $$\begin{aligned} \langle x_i(t) x_j(t-\delta ) \rangle _{data} = \frac{1}{T-\Delta } \sum _{t=\Delta }^T x_i(t) x_j(t-\delta ), \end{aligned}$$

for $i,j=1,....,N$ (with *N* as the number of zones we took into consideration) and $\delta =0,....,\Delta$. Another common choice is to fix the average value of all the system variables. We took $\langle x_i \rangle _{data} = \frac{1}{T-\Delta } \sum _{t=\Delta }^T x_i(t)$ and $\langle x^2_i \rangle _{data} = \frac{1}{T-\Delta } \sum _{t=\Delta }^T x^2_i(t)$.

From these, we obtain the equation for the probability:

10 $$\begin{aligned} P(x(t), \dots , x(t-\Delta )) = \frac{1}{Z} \exp \left[ -\sum _{t=\Delta }^T \sum _i a_i x_i^2(t) + \sum _{t=\Delta }^T \sum _i h_i x_i(t) + \sum _{t=\Delta }^T \sum _{\delta =1}^\Delta J_{ij}^\delta x_i(t)x_j(t-\delta ) \right] , \end{aligned}$$

where $a_i$ is the *i*-th component’s standard deviation, $h_i$ is its mean and $J_{ij}^\delta$ are the time shifted interactions.

Writing $v_i(t) = h_i + \sum _\delta J_{ij}^\delta x_j(t-\delta )$ one can obtain:

11 $$\begin{aligned} P(x(t), \dots , x(t-\Delta )) = \frac{1}{Z} \prod _{t=\Delta }^T \exp \left[ -\sum _i a_i x_i^2(t) + \sum _i v_i(t) x_i(t) \right] . \end{aligned}$$

### Time series forecasting

We trained the model for 100000 steps using several hyperparamenters ($\Delta = \{24,36,48,72\}$ and the regularization factor $\lambda =\{0.001,0.004,0.005,0.006,0.01\}$, see Table 1), defined in 24. For more detail about the parameters, we refer to the "Methods" section.

To quantify the predictive capabilities of our models, we used the *Mean Absolute Error (MAE)*, the *Mean Squared Error (MSE)* and the *Coefficient of Determination*, also known as $R^2$^45^.

**Table 1:** $R^2$ values over the test set w.r.t. $\Delta$9 and $\lambda$ 24 values, resulting from 100000 steps of training.

$\Delta$	$\lambda$	$R^2$
24	0.001	0.855316
24	0.004	0.854723
24	0.005	0.860069
24	0.006	0.853689
24	0.01	0.832542
36	0.001	0.854458
36	0.004	0.85442
36	0.005	0.853946
36	0.006	0.853447
36	0.01	0.832672
48	0.001	0.860072
48	0.004	0.861741
**48**	**0.005**	**0.869466**
48	0.006	0.861626
48	0.01	0.833099
72	0.001	0.85655
72	0.004	0.859633
72	0.005	0.867582
72	0.006	0.859632
72	0.01	0.858497

Values for the best model are in bold.

**Figure 2:**
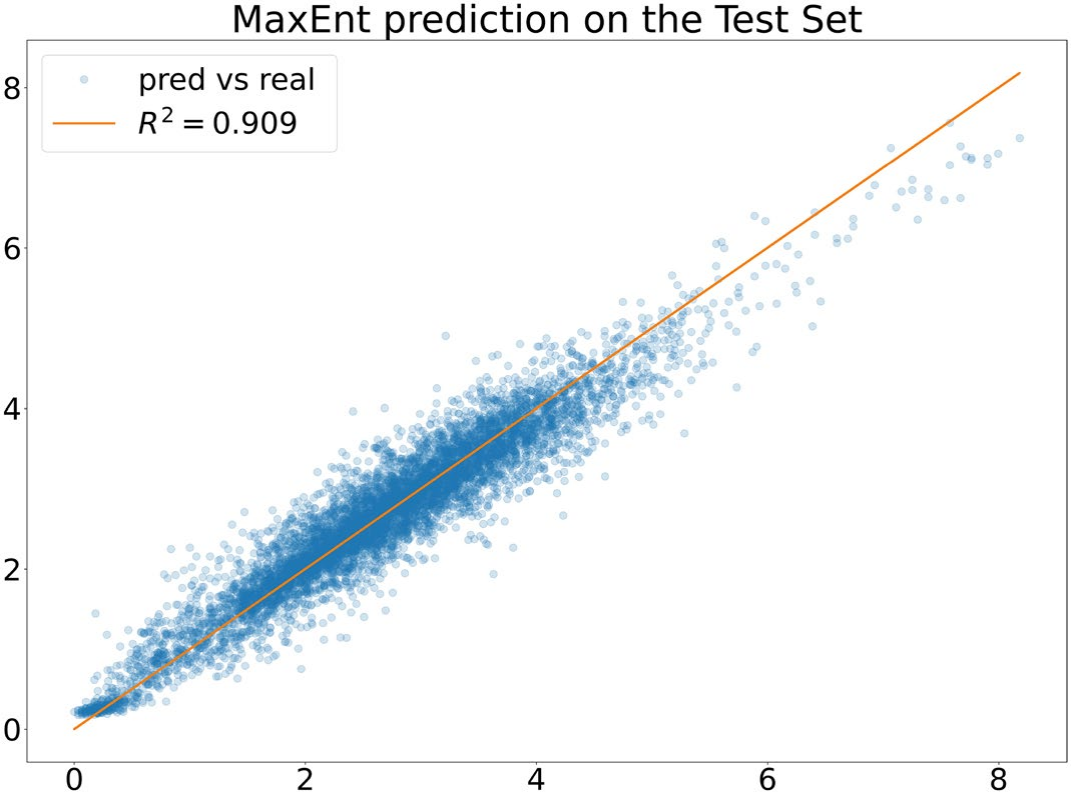
$R^2$ MaxEnt prediction over the test set. To have $R^2=1$, the point should align on the bisector line, having a perfect predictor.

We typically find good predictive capabilities on the test set ( as in Fig. 2, and the exact values for each predictor are found in Fig 1). For $\Delta = 48$ (exactly the day-periodicity) and $\lambda =0.005$, we find our model’s best performance, so we will use these values, training the model for 3,00,000 steps.

To assess the predictive power of our approach against more complex models, we compared it with standard statistical inference and Machine Learning methods. In particular:

1. a **SARIMA** model^31^: we performed a grid search over the parameters using *AIC*^46^ as metrics; the best predictive model is, referring to standard literature on the topic, $\{p=2,d=0,q=1,P=2,D=1,Q=2,m=48\}$. In Table *R*1 in Supplementary Information, we show the table of the grid search.
2. **NN** and **LSTM**: each is composed of $N_{layers}$ hidden layers with a number of nodes $N_{nodes}=\{64,128,256,512\}$. Between the layers, we performed dropout techniques and standard non-linear activation functions (ReLU). A linear layer is added at the end of the *LSTM* layers. Performing a grid search over the other Machine Learning hyperparameters, the models with $N_{layers}=3,4$ and $N_{nodes}=128,256$ have been selected, giving similar results in terms of forecasting ability. The other hyperparameters tuned are learning rate (grid values: $\Bigl \{ 0.001,0.0005,0.0001,0.0001,0.00005 \Bigl \}$, set to 0.0001), the maximum number of epochs during the training process (grid values: $\Bigl \{ 30000,20000,$ $10000,5000 \Bigl \}$, set to 10000), percentage of dropout between the layers (grid values: $\Bigl \{ 0.4,0.3,0.2,0.1 \Bigl \}$, set to 0.2 or 0.3), lambda values in regularization techniques (for our case, using either drop out or regularization gave the same results).

**Figure 3:**
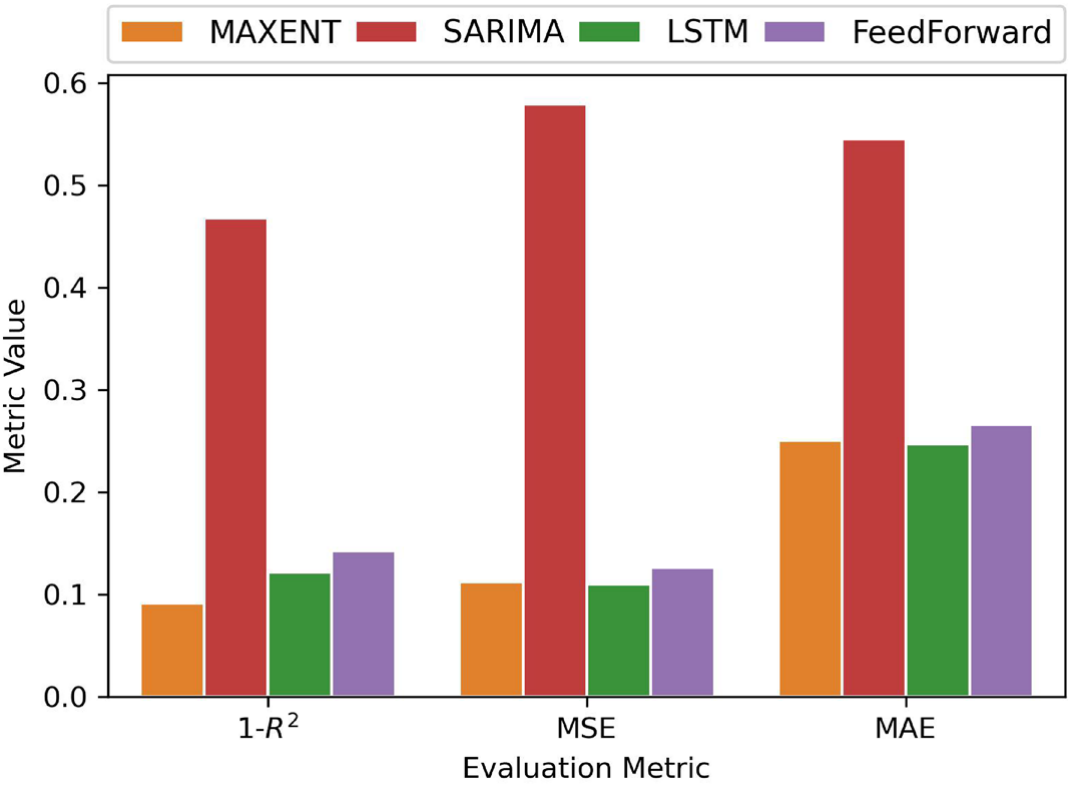
Model comparison using different metrics. On the x-axis, the different metrics used to evaluate the models. On the y-axis, their respective values. For each metric, low values indicate good model predictions of the activity. We see similar results between the MaxEnt and Deep Learning models.

**Figure 4:**
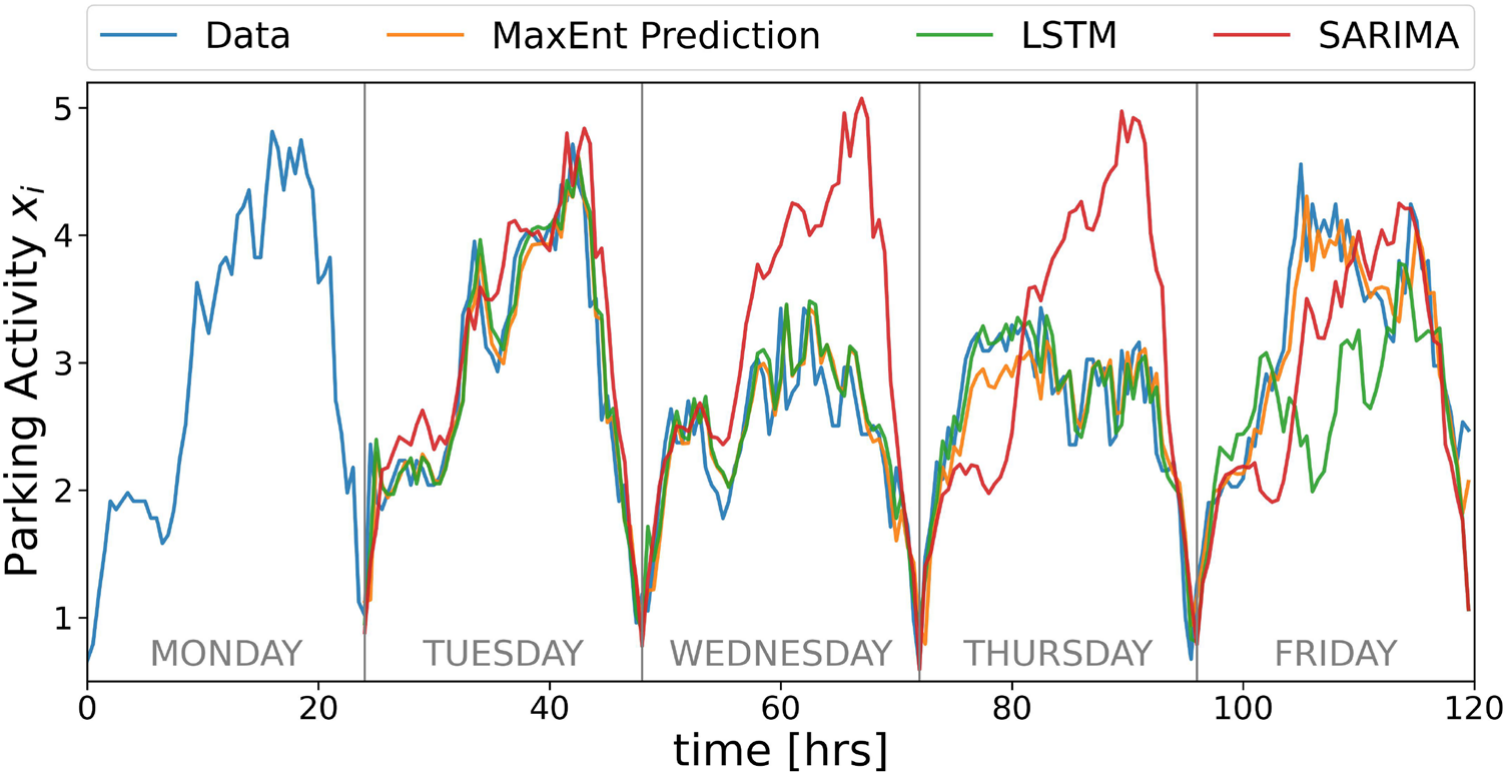
Comparison of the parking activity data (blue) with the prediction of the three models. We can observe that SARIMA models are good predictors of periodicity, but the other models perform better when the change between one period and another is high.

Figure 3 shows that our model outperforms SARIMA and performs as well as the Machine Learning models. Since the LSTM model always performs slightly better than the FeedForward, we will use the first as a reference. Figure 4 shows this in more detail: SARIMA fails to reproduce variations, and it is mostly focused on seasonality. Regarding the LSTM model, we observe in Fig. 4 that it completely fails in catching the different behavior of the activity signal of the selected area on Friday, whereas the MaxEnt prediction correctly catches the different pattern.

Our model works as well as Neural Networks but with orders of magnitude of fewer parameters. In Supplementary Information we present the plot of the number of parameters for the best model for each technique (Fig. *R*3, page 4).

In Fig. *R*2 we show data and results for the cities of Rome, Florence, and Turin (Supplementary Information, page 3). In Fig. *R*4, we show the results in a multiple-horizon framework: we don’t focus on short-range predictions; instead, we predict multiple steps of the time series (Supplementary Information, page 4).

### Extreme events prediction

As already pointed out, prediction is a possible application of our procedure, and we compared the results with state-of-the-art forecasting techniques. Another possible application is the detection of outliers in the data. We see from Fig. 1 that our car-sharing data exhibits regular patterns on different days. These patterns can be disturbed by many different external events, influencing the demand for car-sharing vehicles and the city’s traffic patterns. Suppose we compute the log-likelihood from equation (17), restricted to one of the disturbed days. In that case, we expect it to be particularly low, implying that the model has lower predictive power than usual. Hence, we can use the log-likelihood ${\mathscr {L}}$ from equation (17) as a score in a binary classification task. After training the model with a specific parameter choice, we consider a set of days in the test dataset, $d_1 \dots d_D$. Each one is a set of consecutive time steps: $d_i = [t^{d_i}_1, t^{d_i}_2]$ with $t^{d_i}_1 < t^{d_i}_2$. The log-likelihood of a single day is just

12 $$\begin{aligned} {\mathscr {L}}_{pseudo}(d_i) = \frac{1}{t^{d_i}_2 - t^{d_i}_1-\Delta } \sum _{t= t^{d_i}_1-\Delta }^{t^{d_i}_2} \log P(x(t) | x(t-1), \dots , x(t-\Delta )). \end{aligned}$$

We can now assign a label $L(d_i)=0$ if $d_i$ as a standard day and $L(d_i)=1$ if it is a day where a specific external event occurred. We considered two types of external events:

1. Weather conditions: on that day, there was fog, a storm, or it was raining.
2. Strikes: that day, there was a strike in the city that could affect car traffic. We considered taxis, public transport, and general strikes involving all working categories.

**Figure 5:**
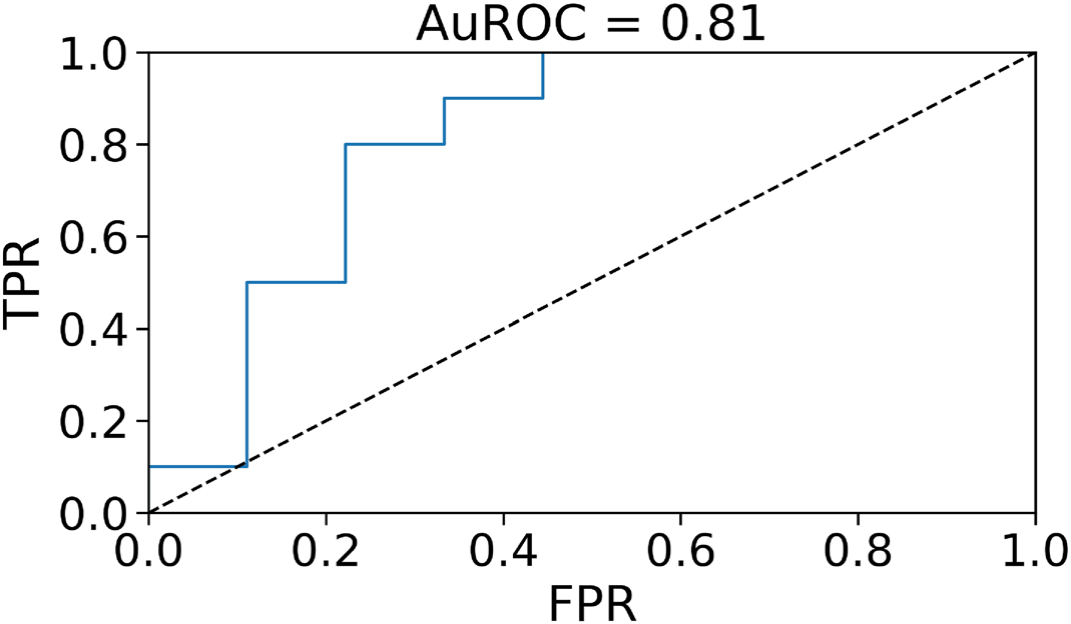
ROC curve for the detection of outliers, i.e. bad weather conditions and strikes, for $\lambda =0.006$ and $\Delta =48$.

We had $50\%$ of days labelled as 1 among the days in the test dataset. In Fig. 5, we show the ROC curve for the classification of such days, using the log-likelihood trained with the parameters $\{\Delta =48,\lambda =0.006\}$. The area under the ROC curve (AuROC) is 0.81, indicating that ${\mathscr {L}}$ is a good indicator of whether an external event occurred on a specific day.

## Discussion

In this work, we have addressed the problem of building statistical and machine learning models to predict mobility patterns within the Milan Metropolitan Area. We focused on predicting how many car-sharing vehicles are parked within a specific area at different times. To do this, we analyzed a longitudinal dataset of parking events collected from the APIs of the Italian car-sharing company *Enjoy*. The processed data consisted of a collection of time series representing the number of parked vehicles in a given area at a given time.

To predict the evolution of these time series, we leveraged the Maximum Entropy (ME) modelling framework, which requires the identification of relevant observables from the data and the use of these observables to define probability density functions depending on some parameters.

Maximum Entropy modelling has proven to be very effective at studying and determining the activity patterns inside the city (Fig. 3).

We compared our model with other models built explicitly for time series forecasting. In particular, we used a SARIMA model, a Feed-Forward Neural Network, and a Long Short-Term Memory Neural Network.

Maximum Entropy models outperformed SARIMA models with reference to all the metrics used in the evaluation. Our model is as predictive as LSTMs Neural Networks but uses two orders of magnitude fewer parameters, and it is possible to use our model to perform different kinds of predictions.

Finally, we also used the statistical model to identify extreme events affecting urban traffic, such as bad weather conditions or strikes.

In conclusion, we contribute to the literature on aggregated data human mobility studies in different ways. We present a new statistical model that outperforms state-of-the-art techniques used in aggregated time-series forecasting tasks. Its predictive ability is comparable only to massive LSTM models. However, LSTM models need to be trained on a hyperparameter space that is orders of magnitudes larger than our MaxEnt model and lacks a clear interpretation. In fact, the parameters of LSTM models can no longer be interpreted as effective interactions between different zones. Our model can select the observables that are important for car-sharing mobility.

For example, having defined that time-lagged correlations are essential observables, we add them to the model and find out that they are sufficient to explain time variations and extreme events.

Our finding that linear correlations are a key ingredient to predict mobility patterns is an important result that requires additional investigations.

Moreover, the benchmarked models are built *ad hoc* for forecasting tasks. However, as we showed above, our trained model can be applied to other problems like extreme event prediction and multiple-horizon forecasting. In the case of methodologies other than ours, new models have to be trained to deal with new tasks, thus losing generality and increasing computational costs.

In this article, we compare not only predictive abilities but also computational costs^47^ and interpretability.

In Supplementary information, we show the prediction ability for three additional cities: Rome, Florence, and Turin, showing also that our approach is general.

Given our results, several research directions could be taken to improve and extend the results:

- A more extensive study on the effects of seasonality could help in building better models. Season-dependent models could be built by taking into account larger datasets.
- Evaluate how the prediction ability of the Maximum Entropy models is dependent upon the city’s structure or the resolution of the time series.
- Entangling mobility patterns with other city indicators, such as socio-political disparities and economic indicators, can lead to a better model that depicts human distribution.
- The inclusion of non-linear interaction in the ME models could be difficult if made by defining standard observables. Instead, *hidden variables* approaches could be taken into account, e.g. Restricted Boltzmann Machines^48^.
- The ME models could be adapted to perform other anomaly detection tasks, i.e. identifying parts of the time series which are not standard fluctuations of the system^49,50^.
- Its prediction ability could be benchmarked with more models^30^, highlighting the pros and cons of the different techniques.
- The possibility of multiple horizon forecasting could be expanded and benchmarked with models bulti for long-range predictions.

## Methods

### Dataset

The dataset used in this work is a log of the locations of cars belonging to Enjoy, a famous Italian car-sharing service. We report all the details about collecting and handling data in the section Data Availability.

We collected the data recording all the movements of Enjoy cars within the cities of Milan, Florence, Turin, and Rome in 2017. In the rest of this article, we will focus mainly on parking events, but our methods and analyses could also be applied to study the volume of travel events. One aim of the work was to predict the number of vehicles parked at a given time in each city district. To this end, we divided the service area into the different municipalities (we will call them *zones*). We considered different tessellation strategies (e.g., using hexagons with a 900*m* side), but the municipality division provides more statistically stable training data, and thus more precise models.

For each type of event (parked car) we collected, we defined the *activity* of a zone as the number of events (i.e., cars parked) occurring there in a certain fixed amount of time $\delta t$. Indicating each zone with the index *i*, we obtained for each of them a discrete time series $x_i(t)$ representing the *activity* of the zone *i* in the time frame $[t, t+\delta t]$. We will indicate with $t=T$ the last time frame observed in the data. In the following, we will use a $\delta t=1800\,$s, corresponding to 30 minutes. We chose this time bin width to have a stable average activity $\langle x_i(t)\rangle$ and a stable variance $\sigma ^2(x_i(t))$ over all the zones. Indeed, narrower or larger time bins feature unstable mean or variance in time: the $30-minutes$ binning is significantly stable throughout the observation period. This characteristic helps the model generalize and predict better as the distributions of the modelled quantities do not change too much in time.

The model we propose in this work is defined for real-valued time series. Having a real-valued model is not a trivial choice since it allows us to derive an exact analytical expression for its log-likelihood. However, the $x_i(t)$ activity we have defined so far belongs to ${\mathbb {N}}$ by definition. For this reason, we defined another kind of variable dividing the activity by the standard deviation:

13 $$\begin{aligned} z = \frac{x}{\sigma }, \end{aligned}$$

where *x* is the original activity data, and $\sigma$ is the standard deviation of the activity in time. In this case, for the population, we consider the *typical day*: the std is computed for the same time bin for every day. So, Eq. (13) gets into:

14 $$\begin{aligned} z_i(t)= \frac{x_i(t)}{\sigma _{i}(t_{\delta t})}, \end{aligned}$$

where $t_{\delta t} = t\mod (\delta t)$ is the integer division of t by the $\delta t$ time bin width, $\mu _i(t_{\delta t})$ is the average over all the activities of the zone *i* at times with the same value of $t \mod \delta t$, and $\sigma _{i}(t_{\delta t})$ is their standard deviation. To keep the notation formalism, we will indicate $z_i(t)$ with $x_i(t)$, keeping in mind that *x* now refers to a *normalized activity*.

From now on, we will work on the normalized $x_i(t)$: these indicate how much a given area is more “active”—i.e., has more cars parked - concerning the average volume of that time bin *t* (typical day activity) by weighting this fluctuation with the standard deviation observed for that zone-hour volume. In this way, we can compare the signal of areas with high fluctuations and high activities with less frequented areas around the city.

The final step when processing data for inference and Machine Learning is to divide the data into a *train set* and a *test set*. Taking the time series representing all the Zones’ activity in a working year, we split the dataset, $80\%$ of it for the train and $20\%$ for the test set. We trained the models using the first dataset and tested their precision on the second one to check their ability to generalize to unseen data.

As a final remark, we indicate with *t* the time bin of activity, i.e. $x_i(t)$ indicate the activity of the zone *i* in a time range $[t \delta t, (t+1)\delta t]$.

### Pseudo-log-likelihood maximization

The Boltzmann probability is defined over the whole time series of all the zones, i.e.

15 $$\begin{aligned} P \Big ( x(t), x(t-1),...,x(t-\Delta ) \Big ) \propto \exp \Big ( {\textbf{H}}(x(t), x(t-1),...,x(t-\Delta )) \Big ). \end{aligned}$$

From this, it is straightforward to define the conditional probability of one-time step *x*(*t*) concerning all the previous ones:

16 $$\begin{aligned} P(x(t) \mid x(t-1),...,x(t-\Delta )) = \frac{P \Big ( x(t), x(t-1),...,x(t-\Delta ) \Big )}{P \Big ( x(t-1),...,x(t-\Delta ) \Big )}. \end{aligned}$$

Using equation (8), we can define the Pseudo-Log-Likelihood as:

17 $$\begin{aligned} {\mathscr {L}}_{pseudo} = \frac{1}{T-\Delta } \sum _{t=\Delta }^T \log P(x(t) | x(t-1), \dots , x(t-\Delta )). \end{aligned}$$

Here, using Eq. (16) and substituting the functional form of the two total probabilities, we obtain

18 $$\begin{aligned} P(x(t) | x(t-1), \dots , x(t-\Delta )) = \prod _i \frac{1}{Z_i(t)} \exp ( -a_i x_i^2(t) + x_i(t) v_i(t)), \end{aligned}$$

with

19 $$\begin{aligned} \begin{aligned}{}&Z_i(t) = \frac{1}{\sqrt{a_i}} \exp \left( \frac{v_i(t)^2}{4a_i} \right) \int _{-\infty }^{v_i(t)/2 \sqrt{a_i}} dz e^{-z^2} = \frac{1}{\sqrt{a_i}} \exp \left( \frac{v_i(t)^2}{4a_i} \right) I\left( \frac{v_i(t)}{2 \sqrt{a_i}} \right) , \\&v_i(t) = \sum _{n=1}^{d} \sum _\delta (J_n ^\delta x^n(t-\delta ))_i + h_i. \end{aligned} \end{aligned}$$

Substituting in eq. (17), we get:

20 $$\begin{aligned} {\mathscr {L}}_{pseudo} = \frac{1}{T-\Delta } \sum _{t=\Delta }^T \sum _i -a_i x_i^2(t) + x_i(t) v_i(t) - \log Z_i(t) \end{aligned}$$

and we can calculate the gradients of the ${\mathscr {L}}_{pseudo}$ with reference to the parameters:

21 $$\begin{aligned} \begin{aligned}{}&\frac{\partial {\mathscr {L}}_{pseudo}}{\partial a_{i}} = \frac{1}{T-\Delta } \sum _t -x_i^2(t) - \frac{\partial \log Z_i(t)}{\partial a_i},\\&\frac{\partial {\mathscr {L}}_{pseudo}}{\partial J^\delta _{ij}} = \frac{1}{T-\Delta } \sum _t x_i(t) x_j(t-\delta ) - \langle x_i(t) \rangle x_j(t-\delta ),\\&\frac{\partial {\mathscr {L}}_{pseudo}}{\partial h_{i}} = \frac{1}{T-\Delta } \sum _t x_i(t) - \langle x_i(t) \rangle , \end{aligned} \end{aligned}$$

where

22 $$\begin{aligned} \langle x_i(t) \rangle = \frac{v_i(t)}{2a_i} + \frac{1}{2 \sqrt{a_i} I(\frac{v_i}{2 \sqrt{a_i}} )} \exp \left( -\frac{v_i(t)^2}{4a_i}\right) \end{aligned}$$

and

23 $$\begin{aligned} \frac{\partial \log Z_i(t)}{\partial a_i} = -\frac{1}{2a_i} + \frac{-v_i^2(t)}{4a_i^2} + \frac{1}{I(\frac{v_i}{2 \sqrt{a_i}} )} \exp \left( -\frac{v_i(t)^2}{4a_i}\right) \left( \frac{-v_i(t)}{4 a_i^{3/2}} \right) . \end{aligned}$$

Since we can compute exactly the gradients and the cost function, the parameters’ inference is relatively easy and does not need an approximation.

Once the parameters of the model have been inferred with some optimisation method, it is possible to use it to predict the temporal evolution of the normalized activities of the system. Given some specific state of the system unit time $t-1$, i.e. $(x(t-1), \dots x(t-\Delta ))$ (past time steps further than $\Delta$ from *t* are not relevant), we can use equation (18) to predict the next step *x*(*t*). Since the probability in (18) is a normal distribution whose average is entirely defined by $(x(t-1), \dots x(t-\Delta ))$, the best prediction of $x_i(t)$ is the average of the distribution $\langle x_i(t) \rangle = \frac{1}{2a_i} \left( v_i(t) + \frac{1}{Z_i(t)} \right)$.

In other words, we are using the generative model to make a discrimination task. In this way, it is possible to compare this model with standard machine learning ones by checking their precision in predicting the time series. To avoid over-fitting, we used L1-regularization^51,52^. An in-depth description of the technique can be found in the references. In practice, the cost function that has to be optimized is:

24 $$\begin{aligned} C(\theta ) = \log P(\theta | X) = \log P( X | \theta ) - \lambda \sum _i |\theta _i| + cost. \end{aligned}$$

The first term of this sum is the Log-likelihood; instead, the second term is the regularization term.

Performing the gradient, we obtain the following:

25 $$\begin{aligned} \frac{\partial C(\theta )}{\partial \theta _i} = \frac{\partial \log P( X | \theta )}{\partial \theta _i} - \lambda sign(\theta _i), \end{aligned}$$

where *sign* is the sign function.

The training curves show no sign of overfitting, as the log-likelihood asymptotically stabilizes for the validation and train sets (see Fig. *R*1 in Supplementary Information).

## Supplementary Information

Supplementary Information.

Supplementary Information.

## Data Availability

The data has been collected via the Enjoy APIs, scraping the information through a client account. We obtained information about the area of service (i.e., the limit where people can start and end their ride), the location of vehicles, and the points of interest (POIs) related to the service (such as fuel stations and reserved parking) from the API endpoints. The endpoints used have base URL https://enjoy.eni.com/ajax/ and the following endpoints: retrieve_areas for the area, retrieve_vehicles for cars, and retrieve_pois for points of interests. Through the scraping procedure, we collected a series of events that are divided into two categories: parking events and travel events of each Enjoy car we could observe. Each event comes with information such as the car’s number plate, the time at which the event occurred (with the precision of seconds), and the latitude and longitude of the parking spot in the case of parking events. Starting and arrival points in the case of travel events are also recorded. We included the activity for Milan in Supplementary Information files: the dataframe format shows the $30-min$ binning for each zone ($ZONE\_IDX$) for each working week ($WORKING\_WEEK$). We also included the shapefiles for the municipalities and files for weather conditions and strikes. The datasets for the other cities used and analyzed during the current study are available from the corresponding author upon reasonable request.
