# Supplementary material for: A maximum entropy approach for the modelling of car-sharing parking dynamics
Source: Sci Rep. 2023 Feb 21;13:2993. doi: 10.1038/s41598-023-30134-9 (PMC9945450; doi:10.1038/s41598-023-30134-9)
Supplement: Supplementary file 1 — Supplementary Information. [file 41598_2023_30134_MOESM1_ESM.pdf]

A Maximum Entropy approach for the Modelling of Car-Sharing Parking Dynamics

Additional Information

Table R1: Grid search over the SARIMA parameters

p	d	q	P	D	Q	m	AIC
1	0	1	0	1	1	48	inf
0	0	0	0	1	0	48	1243.245
1	0	0	1	1	0	48	359.487
0	0	1	0	1	1	48	inf
0	0	0	0	1	0	48	1241.606
1	0	0	0	1	0	48	615.697
1	0	0	2	1	0	48	285.295
1	0	0	2	1	1	48	270.777
1	0	0	1	1	1	48	279.520
1	0	0	2	1	2	48	237.403
1	0	0	1	1	2	48	inf
0	0	0	2	1	2	48	inf
2	0	0	2	1	2	48	236.616
2	0	0	1	1	2	48	inf
2	0	0	2	1	1	48	270.899
2	0	0	1	1	1	48	280.519
2	0	1	1	1	2	48	inf
2	0	1	2	1	1	48	273.366
2	0	1	1	1	1	48	276.647
1	0	1	2	1	2	48	237.017
2	0	2	2	1	2	48	237.784
1	0	2	2	1	2	48	236.860
2	0	1	2	1	2	48	233.925

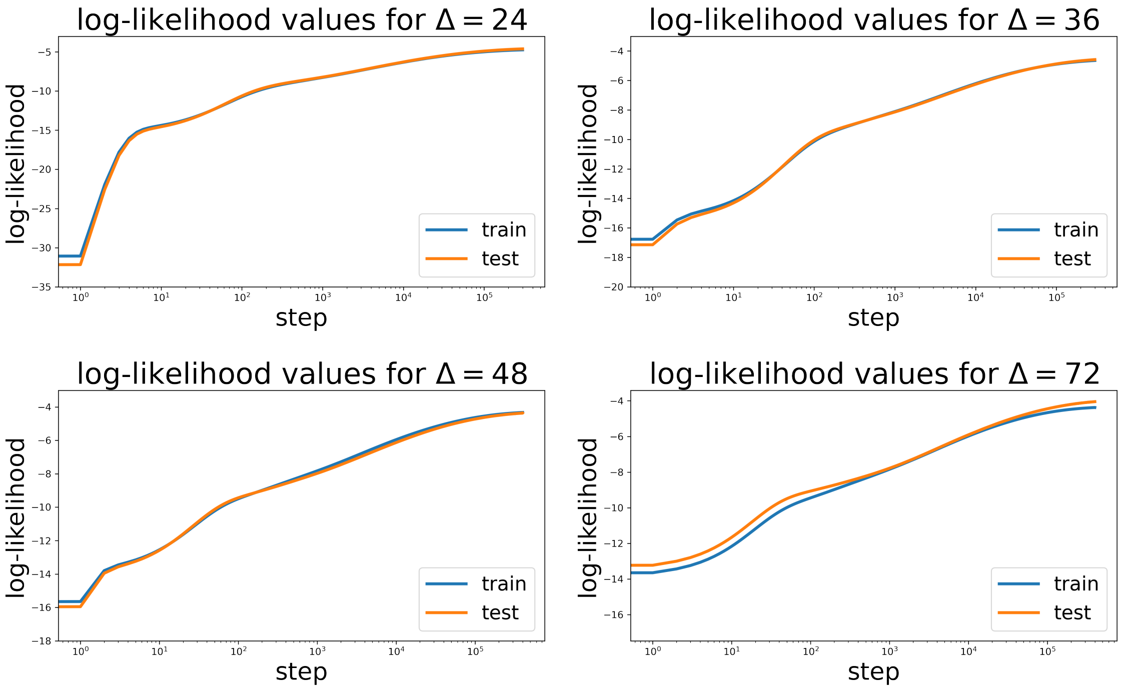

Figure R1: Log-Likelihood training for different values of Δ.

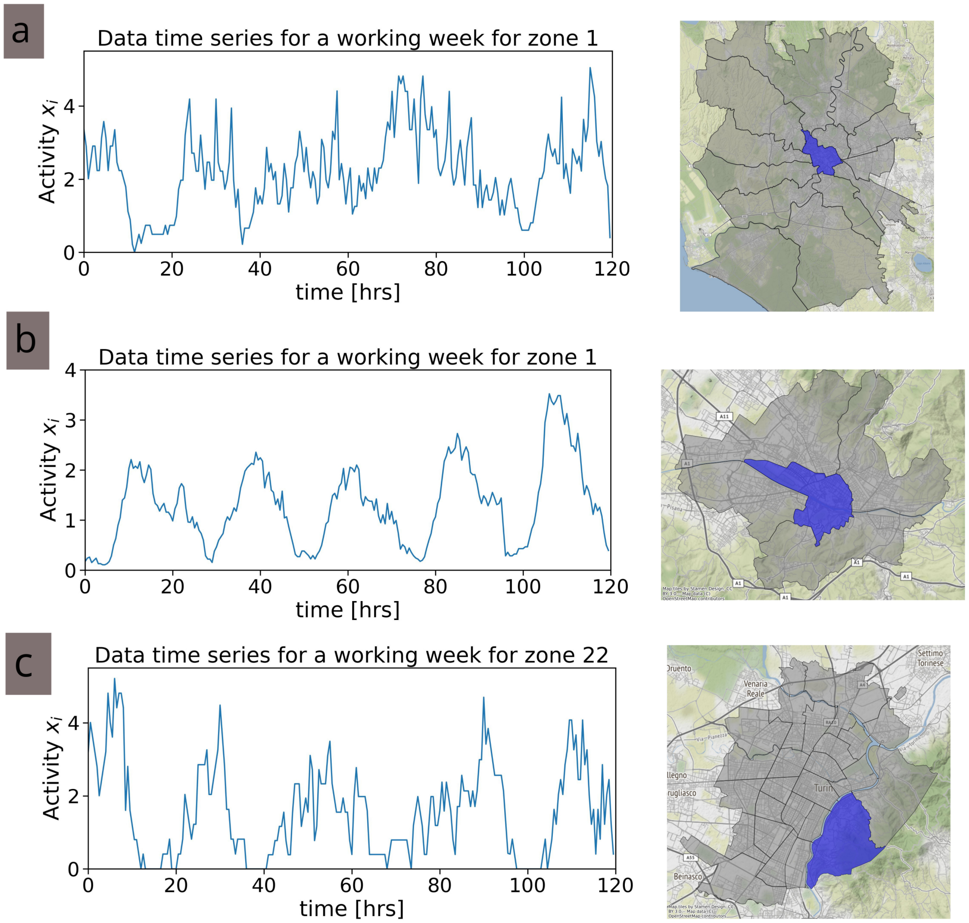

Figure R2: Time series activity data for the cities of Rome (a) having $R^2 = 0.816849$, Florence (b) having $R^2 = 0.748687$ and Turin (c) having 0.727507 .

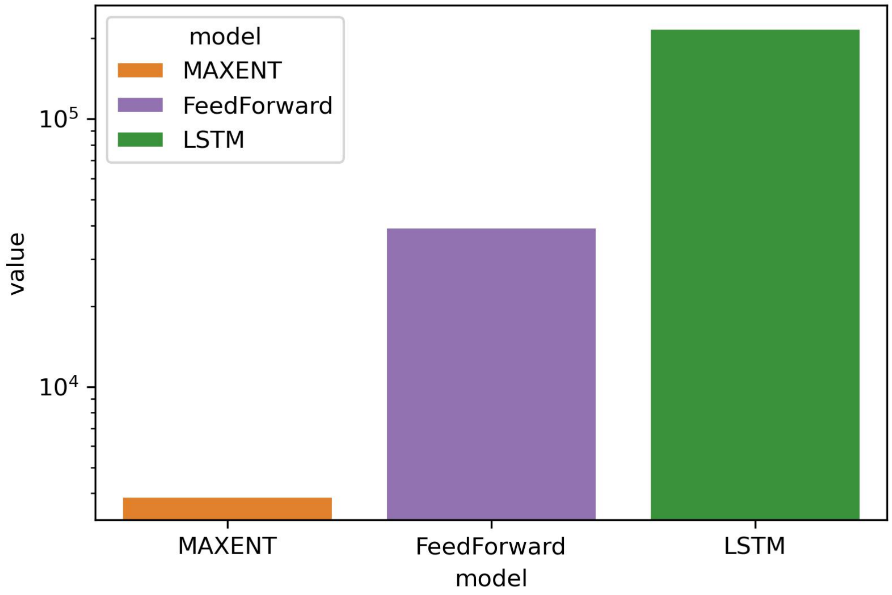

Figure R3: Number of parameters to be learned by the different models.

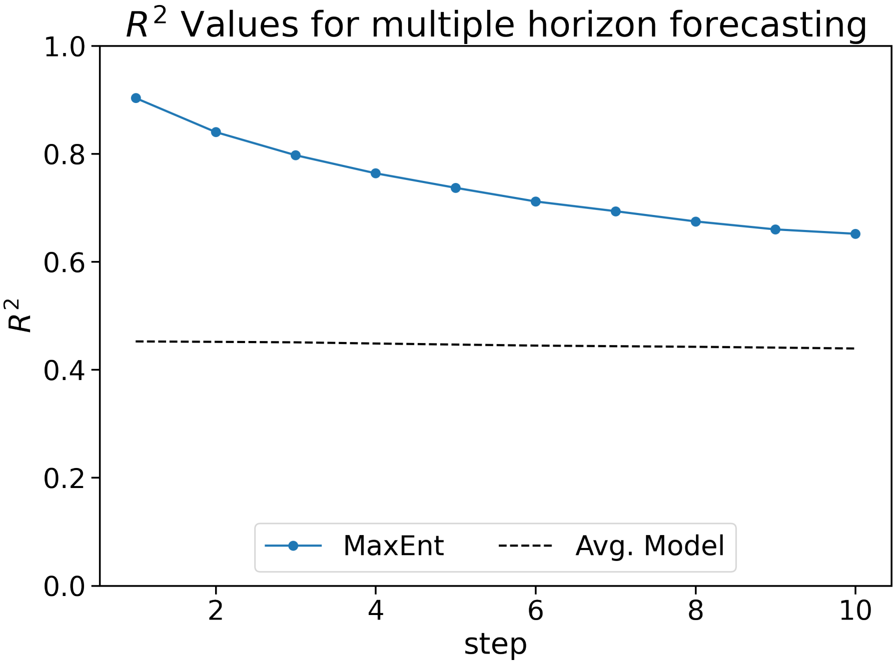

Figure R4: R^2 value for multiple horizons of forecasting, using the MaxEnt model of the paper. Its predictive ability is benchmarked using the R^2 values produced from the mean of the distribution.
